# Combatting Sedentary Lifestyles: Can Exercise Prescription in the Emergency Department Lead to Behavioral Change in Patients?

**DOI:** 10.7759/cureus.7071

**Published:** 2020-02-21

**Authors:** Fiona Milne, Kalen Leech-Porter, Paul Atkinson, David Lewis, Jacqueline Fraser, Stephen Hull

**Affiliations:** 1 Internal Medicine, Queen's University, Kingston, CAN; 2 Family Medicine, Dalhousie Family Medicine, Halifax, CAN; 3 Emergency Medicine, Saint John Regional Hospital, Saint John, CAN; 4 Emergency Medicine, Dalhousie University, Halifax, CAN; 5 Endocrinology, Saint John Regional Hospital, Saint John, CAN

**Keywords:** exercise prescription, emergency medicine, health promotion

## Abstract

Introduction

Emergency department (ED) patients with chronic disease are known to benefit from exercise; however, there are few studies examining the prescription of exercise in the ED. We asked, is exercise prescription in the ED feasible and effective?

Methods

In this pilot prospective block randomized trial, consented patients were divided into control and intervention groups. The control group received routine care. The intervention group received combined written and verbal prescriptions for moderate exercise of 150 minutes/week. Both groups were followed up by phone at two months. The primary outcome was achieving 150 minutes of exercise per week. Secondary outcomes included change in exercise and differences in reported median weekly exercise.

Results

Follow-up was completed for 23/28 patients (11 control; 12 intervention). Baseline reported median (with interquartile range) weekly exercise was similar between groups: control 0 (0-0) minutes, intervention 0 (0-45) minutes. There was no difference between groups for the primary outcome at two months (control 3/11; intervention 4/12, relative risk [RR] 1.33 (95% confidence interval [CI] 0.38-4.6; p=1.0). There was a significant increase in median exercise from baseline in both groups, but no difference between the groups (control 75 (10-225) minutes; intervention 120 (52.5-150) minutes; NS). A post hoc comparison of patients actually receiving intervention vs. no intervention revealed a significant increase in patients meeting the primary outcome (no intervention 0/8; intervention 7/15, RR 2.0 (95% CI 1.2-3.4); p=0.05).

Conclusion

The improvement seen in patients receiving the exercise prescription intervention, and the increase in reported exercise in both groups suggests that exercise prescription for ED patients may be beneficial.

## Introduction

Frequent physical activity is known to reduce mortality and comorbidities associated with chronic illness and can have positive effects on mental well-being. Exercise prescription is a relatively new practice, but its implications on systemic health outcomes cannot be ignored. Exercise has been shown to be therapeutically effective in many diseases and physical activity is beneficial in reducing risk factors and disease progression for many chronic illnesses, including type 2 diabetes and hypertension and cardiac disease [1-3]. In the primary care setting, exercise counseling has been shown to increase physical activity among patients [4]. Exercise counseling and prescription could potentially decrease patient comorbidities, and prevent adverse events such as falls, which could decrease health care burden and improve patient outcomes [5]. Walking, which is accessible to the majority of patients, has been shown to be effective in most chronic health conditions. A systematic review of Canada's physical activity guidelines found evidence supporting a goal of five days of 30 minutes moderate-intensity exercise per week [6].

There are few reports of exercise prescription in the emergency department (ED). While emergency physicians face time constraints, their ability to play a role in advocating lifestyle modifications is being increasingly documented, especially for patient populations where access to reliable primary care is unavailable [7]. A recent study by Soegtrop et al. reported that 62.7% of the Canadian emergency physicians counsel their patients in other forms of preventative medicine, such as smoking cessation and safe sex, and have shown effectiveness [8]. Our pilot study aims to study the feasibility and estimate the effect size of exercise prescriptions from the ED. 

## Materials and methods

Study design and setting

This was a pilot prospective comparative study, utilizing block randomization, looking at the role of exercise prescriptions in the ED. The study took place at a provincial tertiary hospital ED in New Brunswick, Canada. Participation was voluntary and confidential. The study was approved by the Horizon Health Network Research Ethics Board in April 2016 (REB 2016-2281). This work has previously been presented as an abstract at the Saint John Regional Hospital, Saint John, NB, March 24, 2017 (http://sjrhem.ca/wp-content/uploads/2017/03/fiona.pdf).

Population

Eligible study patients met the following inclusion criteria: age 30-70 years old, not already exercising 150 minutes per week. Patients were excluded if the physician felt there were any medical contraindications to exercise (e.g. unstable angina or significant fall risk). Patients were also excluded if they were admitted to the hospital.

Materials and procedure

Potential study participants were identified at triage and after informed consent, were randomized to either control (standard care) or the intervention group, based on the week of presentation to the ED. Intervention group patients were provided with a verbal prescription and a standardized provincial written exercise prescription to exercise at a moderate intensity for 150 minutes per week. All participants completed a discharge survey in the ED, with questions on both demographics and baseline exercise habits. At two months, they completed a follow-up telephone or written survey.

Outcomes

Our primary outcome was the number of patients who self-reported that they met the target of 150 minutes per week of moderate exercise comparing pre- and post-surveys. Our secondary outcome was the self-reported change in minutes of exercise per week per patient. 

Data analysis

Survey responses for each group were summarized with descriptive statistics. Continuous variables (e.g. age) were summarized with means and standard deviations or medians with interquartile ranges. Categorical variables (e.g. gender) were summarized with frequencies and percentages. 

A linear regression model was used to determine the mean difference between the control and intervention groups and mean differences from pre-intervention to post-intervention. The dependent variable was the number of minutes of exercise activity. The independent variables consisted of a between-groups factor (intervention group) and a within-groups factor (measure; pre/post-intervention). Alpha level for statistical significance was set at 0.05 for a sample size of 90 to detect a moderate effect size. Planned comparisons were made between the mean number of minutes of exercise activity pre-intervention and post-intervention for the two groups. In addition to our intention-to-treat analysis, we completed a post hoc analysis to compare actual intervention received.

## Results

Baseline comparison

**Figure 1:**
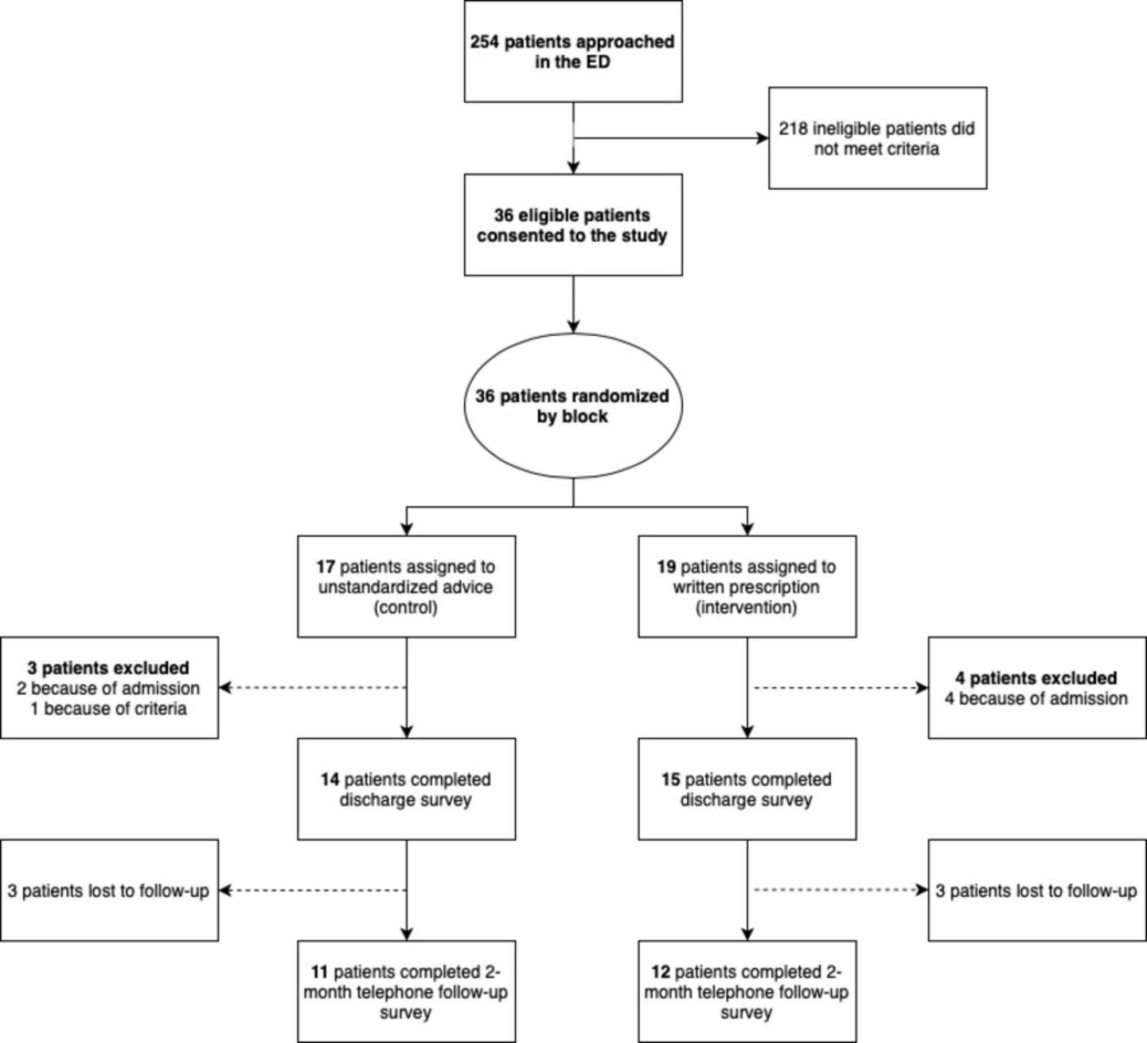
Consort diagram of patient enrollment and eligibility criteria

A total of 36 participants met the inclusion criteria and were consented into the study. Based on block randomization, 17 patients were enrolled in the control group and 19 patients were enrolled in the intervention group. Three control and four intervention patients were later excluded due to admission to hospital, with three patients lost to follow-up in each group (see Figure 1). 

Baseline patient characteristics are shown in Table 1.

**Table 1:** Baseline patient characteristics n: number; HTN: hypertension; T2DM: type 2 diabetes

Patient characteristics		Control	Intervention
Total participants enrolled, n		17	19
Total lost to follow up, n		3	4
Total participants included, n		14	15
Age, year, mean		56	54
Gender, female, n		3	11
Chronic health conditions			
	Hypertension, n	8	5
	Type 2 diabetes, n	1	2
	Both HTN and T2DM, n,	2	2
	Neither HTN nor T2DM, n	3	6
Education			
	Did not complete high school, n	0	1
	High school, n	9	3
	Bachelors/college, n	5	9
	Masters or above, n	0	2
Work			
	Unemployed, n	0	0
	Part-time employment, n	1	2
	Full-time employment, n	6	6
	Retired, n	5	4
	Other, n	2	3

Exercise minutes

**Figure 2:**
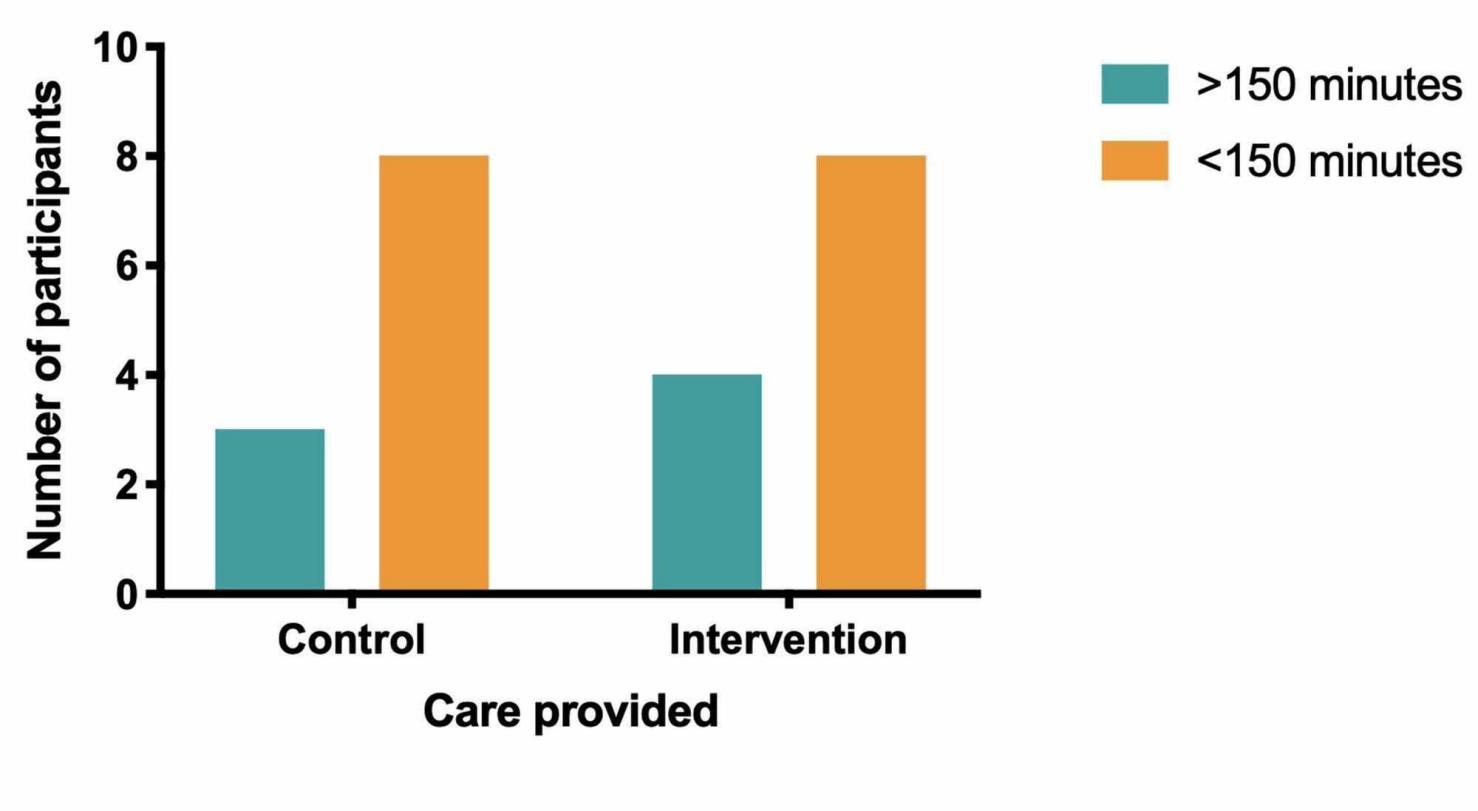
Control and intervention group participants at two-month follow-up–whether or not they meet Health Canada’s guideline of 150 minutes of exercise/week

Follow-up was completed for 23 patients (11 control; 12 intervention). Baseline reported median (with interquartile range) weekly exercise was similar between groups: control 0 (0-0) minutes, intervention 0 (0-45) minutes. There was no difference between groups for the primary outcome of 150 minutes/week at two months (control 3/11; intervention 4/12, relative risk [RR] 1.33 (95% confidence interval [CI] 0.38-4.6; p=1.0; Table 2; Figure 2).

**Table 2:** Outcomes for control and intervention groups n: number; IQR: interquartile range

Group	Control	Intervention
Number completing follow-up	11	12
Reported baseline exercise, minutes/week (median, IQR)	0 (0-0)	0 (0-45)
Number actually receiving exercise prescription or advice, n (%)	3 (27%)	12 (100%)
Achieved primary outcome of 150 minutes/week moderate exercise, n (%)	3 (27%)	4 (33%)
Reported weekly exercise at two-month follow-up, minutes/week (median, IQR)	75 (10-225)	120 (52.5-150)
Secondary outcome-net change in reported exercise, minutes/week (median, IQR)	25 (3-94)	140 (60-225)
Reported weekly exercise increased by at least 30 minutes, n (%)	6 (55%)	9 (75%)

There was a significant increase in median exercise from baseline in both groups, but no difference between the groups (control 75 (10-225) minutes; intervention 120 (52.5-150) minutes; NS).

The net increase in exercise was also monitored as a secondary outcome, where a significant change in exercise was defined as an increase in 30 minutes or greater between baseline and two-month follow-up. There was no difference in net exercise increase between the two groups at two months (control 6/11, intervention 9/12, RR: 0.64; 95% CI 0.282 to 1.45; p=0.4).

**Figure 3:**
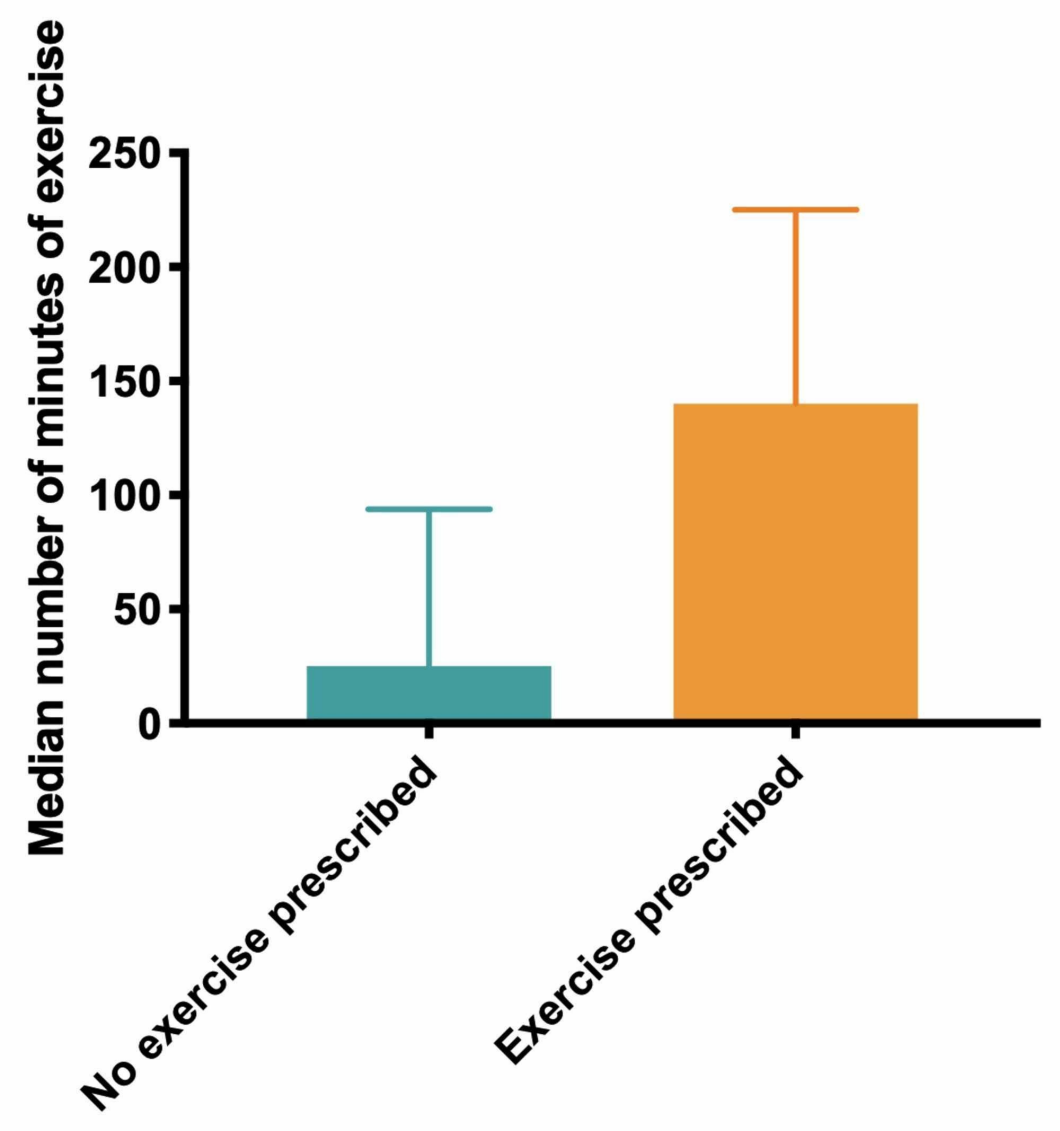
Median number of minutes of exercise reported for patients who did not receive an exercise prescription (no exercise prescription) and those who did (exercise prescription), corrected post hoc for actual care intervention delivered

Further analysis revealed that three control patients had received exercise prescription as part of their routine care. A post hoc comparison of patients receiving any form of exercise prescription and no exercise prescription revealed an increase in patients meeting the primary target of 150 minutes/week (no intervention 0/8; intervention 7/15, p<0.0001; Figure 3). 

In terms of secondary outcomes, the median increase in the no-intervention group was 25 (3-94) minutes compared to 140 (60-225) minutes in the intervention-received group. This difference was determined to be statistically significant (p=0.0134; Mann-Whitney test).

There were no adverse events (falls, injuries) reported from either cohort.

Patient perceptions

Patients in the intervention arm completed additional survey questions on the quality of the exercise prescription they received. The two-month follow-up survey response rate was 80%. Patients rated the detail given in their prescription as 5/5 (±1). The helpfulness of the prescription was rated as 4/5 (±2). Likelihood to continue exercising based on the prescription was rated as 4/5 (±2). Of the 12 participants, 11 felt that exercise should be discussed in the ED either routinely or on a case-by-case basis. One participant felt it should not be discussed at all. 

## Discussion

Despite low recruitment rates, this pilot study has shown that exercise prescriptions warrant further investigation; exercise is inexpensive, relatively low risk, and can, in part, prevent severe health outcomes in diverse patient populations. We found that more participants were meeting Health Canada’s recommended guideline of 150 minutes of aerobic exercise per week at the two-month follow-up if they received an exercise prescription. The prescriptions were also well received from a patient perspective. Participants in this study reported being amenable to having their exercise habits discussed and counseled in the ED, provided that it was relevant to their visit on that day. At follow-up, several participants reported posting their prescription in a visible location, and using it as a reminder to be physically active: this demonstrates that the power of the prescription pad cannot be undermined. Our study is one of the first to measure the behavioral changes associated with exercise prescription.

Much of the literature supporting the evidence behind exercise prescription has focused on health biomarker monitoring and health risk reduction but has not measured patients’ willingness to change, which is an important consideration to make when discussing any major lifestyle change with patients. It is also one of the first studies to explore the potential utility of exercise prescription in the ED. Previous studies have explored exercise prescription in a medical setting where follow-up with patients is expected; these include family medicine, oncology, and physical medicine and rehabilitation. Having shown the feasibility of exercise prescription in the ED, and with some evidence that it could be effective in changing behavior, we call upon emergency physicians and other staff to consider incorporating this approach to combating sedentary lifestyles when addressing health promotion and risk reduction. Although many of the interventions and medications we prescribe on a daily basis have some benefit, few are likely to impact long-term health outcomes as much as effective behavioral change towards increased physical activity [9,10]. As a successful treatment for chronic disease, an effective intervention for reducing obesity, and as a harm reduction tool reducing the risk of death associated with long periods of time sitting, exercise is one of the more cost-effective interventions available for prescription to patients in the ED and other settings [9-11].

The numbers in this study are small. In addition, it is possible that the Hawthorne effect impacted outcomes, as participants in both groups were aware that they would receive a phone call for a follow-up survey on exercise and may have been motivated either to exercise or report increased exercise. 

Though exercise prescriptions may have a potential role in the ED, there are several barriers to implementing this practice, including lack of follow-up and time constraints. Without follow-up, it is potentially more difficult to adjust exercise prescriptions, individualize treatment, and assess safety. However, these same limitations would occur with any medication prescription given from the ED. Furthermore, exercise counseling in the ED would allow for intervention in patients that may not present to the medical system otherwise. This is especially relevant in areas where access to primary care is not universally accessible.

## Conclusions

The provision of exercise prescriptions to ED patients was shown to be feasible. However, our study was underpowered to accurately quantify the impact on subsequent rates of weekly exercise. The reported improvement seen in patients receiving the intervention and the increase in reported exercise in both groups suggests that exercise prescription for ED patients may be beneficial.
